# Sm-like proteins in the pathogenesis of Spinal Muscular Atrophy

**DOI:** 10.1186/2193-1801-4-S1-L53

**Published:** 2015-06-12

**Authors:** Tilmann Achsel, Robin Doms, Claudia Bagni, Fabrizio Renzi

**Affiliations:** VIB Center for the Biology of Disease at the KU Leuven, Leuven, Belgium

**Keywords:** SMA, mRNA regulation, Sm-like proteins

Proximal Spinal Muscular Atrophy (SMA) is caused by an insufficient supply of the Survival of Motor Neurons (SMN) protein. It is characterized by the selective degradation of the alpha motor neurons in the spinal cord. Analysis of the mouse model, which faithfully mimics the SMN insufficiency and the motor neuron phenotype, shows that motor neuron degeneration starts in the axons, specifically at the neuromuscular junctions. SMN is a house-keeping factor that is necessary for the assembly of the seven-membered ring of Sm proteins around the spliceosomal snRNAs and that is therefore required for pre-mRNA splicing. Such a function cannot easily explain the selective motor neuron phenotype. It has been proposed that insufficient supply of the SMN protein affects alternative splicing, especially of U11/U12-dependent introns, in mRNAs that are crucially required in motor neurons. Details, however, remain elusive. In addition to the Sm proteins, there are the like-Sm (LSm) proteins that also form heptameric complexes and participate in various steps of mRNA metabolism. We have previously shown that one such LSm complex is involved in the transport of mRNAs to the neurites, especially the axons of motor neurons. mRNA transport and local protein synthesis is an important mechanism to bring about sudden changes in the proteome at distal regions of the cell. Here, we further explore the role of the LSm proteins in neuronal mRNA regulation and how this could be relevant for the SMA pathology. Interestingly, we find that one such LSm protein is significantly depleted in the SMA mouse model before the onset of the disease, possibly indicating a causal involvement.

